# The "nocebo effect" in drug hypersensitivity diagnosis

**DOI:** 10.1186/2045-7022-4-S3-O9

**Published:** 2014-07-18

**Authors:** Luisa Bommarito, Sabrina Mietta, Gianni Cadario

**Affiliations:** 1 A.O.U. Città della Salute e della Scienza di Torino- Presidio Molinette, S.C. Allergologia e Immunologia Clinica, Italy

## Background

Placebo-controlled oral drug provocation tests (DPTs) are an important step in drug hypersensitivity diagnosis. Administration of placebo (an inert sostance not detectable from drug) is recommended to avoid psychological influences on the interpretation of DPT results, however actually little is known about how to deal a reaction to placebo and how to go further with the diagnostic procedures.

## Methods

A retrospective study of patients who experienced a reaction to placebo during an oral DPT between January 2009 and December 2013 was conducted at the Allergy Unit of Molinette Hospital of Turin. Demographical characteristics of patients, symptoms developed, number of further DPTs and consequences of reactions to placebo on final diagnosis were analyzed.

## Results

55/673 patients (50 females, 5 males, mean age 44.8 years) reacted to placebo during an oral DPT. The main reported symptoms involved mouth and throat (itching, "lump in the throat", sense of constriction) in 45.4% of cases, skin (itching, erythema, urticaria) in 27.3%, respiratory tract (rhinitis, shortness of breath, cough, chest tightness) in 14.5%, gastrointestinal tract (nausea, heartburn) in 12.7%.A not-specified malaise was reported by 21.8% of patients and other symptoms (headache, agitation, paresthesias....) by 7.3% of cases. 16/55 patients refused further tests; 39/55 patients performed further placebo-controlled oral DPTs on different days and 16/39 reacted again to placebo administration, developing symptoms similar to the previous reaction. A true allergic reaction to the suspected culprit drug was observed in 1 patient. Overall a final diagnosis could not be reached in 27/55 patients.

## Conclusions

During oral DPTs patients could develop symptoms typical of anxiety status, but also symptoms that can mimic a true allergic reaction. A first reaction to placebo influenced negatively the diagnostic process and the development of further reactions to placebo. Other studies are needed to identify therapeutic strategies for these patients and to stop the "nocebo effect".

